# ASSOCIATIONS BETWEEN COGNITIVE PERFORMANCE AND PERCEIVED CONTROL: SPOUSAL SUPPORT AS A MODERATING FACTOR

**DOI:** 10.1093/geroni/igae098.2427

**Published:** 2024-12-31

**Authors:** Michael Buxton, Adrian Williams, Erick Carranza, Valerie Luskey, Hannah Apostolou, M Lindsey Jacobs

**Affiliations:** University of Alabama, Tuscaloosa, Alabama, United States; University of Alabama, Tuscaloosa, Alabama, United States; University of Alabama, Tuscaloosa, Alabama, United States; University of Alabama, Tuscaloosa, Alabama, United States; University of Alabama, Northport, Alabama, United States; The University of Alabama, Tuscaloosa, Alabama, United States

## Abstract

Social support can play an important role in cognitive functioning and involves both actual and perceived support. This presentation used data from the 2020 wave of the Health and Retirement Study. The sample consisted of community-dwelling older adults who completed questions of interest (n= 3416). The sample was predominantly female (59.6%) with a mean age of 68.6 years old (SD=10.3). The purpose of this study was (1) to examine the relationship between cognitive performance and perceived constraints on personal control and (2) investigate how perceived social support across relationship categories influenced the nature of this association. Participants who indicated dementia diagnoses were excluded from analyses. To determine the influence of perceived social support on the relationship of interest, moderation analyses were performed with the SPSS macro PROCESS using mean-centered values. Age, years of education, and gender were used as covariates. Perceived social support and cognitive performance had significant effects on perceived constraints. Positive and negative spousal support were the only relationship categories with significant interaction effects. Specifically, as positive spousal support increased, the relationship between cognitive performance and perceived constraints on personal control became weaker (b=-0.14, SE =.007, t(2499) = -2.0, p = 0.048). Increasing negative spousal support strengthened the relationship (b=0.14, SE =.006, t(2499) = 2.4, p = 0.017). Perceptions of spousal collaboration and support seemingly operate to fortify personal sense of control, which may be compensatory. Further research should explore facets of perceived social support and associations with cognitive performance and perceived control in older adults.

